# Checking responses of goal- and sign-trackers are differentially affected by threat in a rodent analog of obsessive–compulsive disorder

**DOI:** 10.1101/lm.050260.119

**Published:** 2020-05

**Authors:** George H. Vousden, Sloane Paulcan, Trevor W. Robbins, Dawn M. Eagle, Amy L. Milton

**Affiliations:** 1 Department of Psychology, University of Cambridge, Cambridge CB2 3EB, United Kingdom

## Abstract

In obsessive–compulsive disorder (OCD), functional behaviors such as checking that a door is locked become dysfunctional, maladaptive, and debilitating. However, it is currently unknown how aversive and appetitive motivations interact to produce functional and dysfunctional behavior in OCD. Here we show a double dissociation in the effects of anxiogenic cues and sensitivity to rewarding stimuli on the propensity to develop functional and dysfunctional checking behavior in a rodent analog of OCD, the observing response task (ORT). While anxiogenic manipulations of perceived threat (presentation of threat-associated contextual cues) and actual threat (punishment of incorrect responding on the ORT) enhanced functional checking, dysfunctional checking was unaffected. In contrast, rats that had previously been identified as “sign-trackers” on an autoshaping task—and therefore were highly sensitive to the incentive salience of appetitive environmental cues—selectively showed elevated levels of dysfunctional checking under a range of conditions, but particularly so under conditions of uncertainty. These data indicate that functional and dysfunctional checking are dissociable and supported by aversive and appetitive motivational processes, respectively. While functional checking is modulated by perceived and actual threat, dysfunctional checking recruits appetitive motivational processes, possibly akin to the “incentive habits” that contribute to drug-seeking in addiction.

Obsessive–compulsive disorder (OCD) is a common and highly debilitating mental health disorder with an estimated lifetime prevalence of 2.3% (Ruscio et al. 2010). It has been traditionally conceptualized as a disorder in which obsessive thoughts provoke extreme anxiety, and the compulsive performance of idiosyncratic rituals provides temporary relief from this anxiety (Rachman 1976, 1997; Salkovskis 1985). One major subtype of OCD involves patients engaging in excessive checking behavior. Checking behavior itself can be functional, but in OCD, the excessive checking shown by patients becomes maladaptive and performed at the expense of other behaviors (Rachman 2002).

The mechanisms underlying excessive checking are a matter of debate (Robbins et al. 2012; Kalanthroff et al. 2016). A prominent view postulates that checking responses occur in response to perceived threats to reduce anxiety (Rachman 2002; Parrish and Radomsky 2010), due to dysfunction in a “security motivation system” that has evolved to detect environmental threats to survival (Szechtman and Woody 2004; Zor et al. 2009). This is hypothesized to be an open-ended motivational system, where the sense of security is generated by an endogenous feeling of “knowing” or “yedasentience” that is deficient in patients with OCD (Szechtman and Woody 2004). There may also be additional failure points in the security motivation system in OCD, consistent with findings that patients with OCD overestimate threat compared to healthy controls (Tolin et al. 2006), and that healthy controls, like patients, show increases in the urge to check when their perceived threat is artificially elevated (Parrish and Radomsky 2011). In addition to threat—including perceived increases in the probability and/or severity of harmful events occurring (Rachman 2002)—checking is also thought to be exacerbated under conditions of uncertainty. Patients with OCD are less tolerant of uncertainty than healthy controls, and this intolerance is related to checking compulsions (Tolin et al. 2003). While uncertainty may itself be aversive, an aversion to uncertainty alone cannot, however, fully explain compulsive checking. Intolerance to uncertainty is also increased in generalized anxiety disorder (Holaway et al. 2006) and major depressive disorder (Gentes and Ruscio 2011), and in neither of these disorders does this intolerance manifest itself as an increase in compulsive checking.

It is important to note that checking behavior itself is not necessarily maladaptive and constitutes a normal part of the behavioral repertoire of healthy people. However, the compulsive checking observed in OCD patients is clearly dysfunctional and maladaptive. We have previously argued (Eagle et al. 2014) for the importance of distinguishing between these two different types of checking—functional (adaptive and healthy) and dysfunctional (maladaptive and detrimental)—to fully understand OCD in psychological and neurobiological terms. Importantly, even though functional and dysfunctional checking are expressed through the same motor behavior, this does not mean that both are driven by the same psychological processes and neurobiological substrates.

To probe the psychological and neurobiological bases of checking, we previously developed a fully translational, rodent-to-human analog of OCD-like checking in rats that allows functional and dysfunctional checking to be assessed independently—the observing-response task (ORT; Eagle et al. 2014; Morein-Zamir et al. 2018). To briefly summarize this task (Fig. 1A), animals are presented with two levers, of which one is unpredictably reinforced throughout the session. Animals can “check” which lever is currently rewarded with an “observing lever press” (OLP) on a separate lever located at the back of the chamber. Checking can be functional—illuminating a stimulus light above the currently rewarded lever—or dysfunctional—when animals continue to respond when the light is already illuminated, which provides no further information or reward. Thus, this task allows a single behavioral response—a checking lever press—to be psychologically and neurobiologically dissociated into functional and dysfunctional components, in a manner that is more readily quantifiable than alternative ethological tasks such as marble burying.

**Figure 1.**
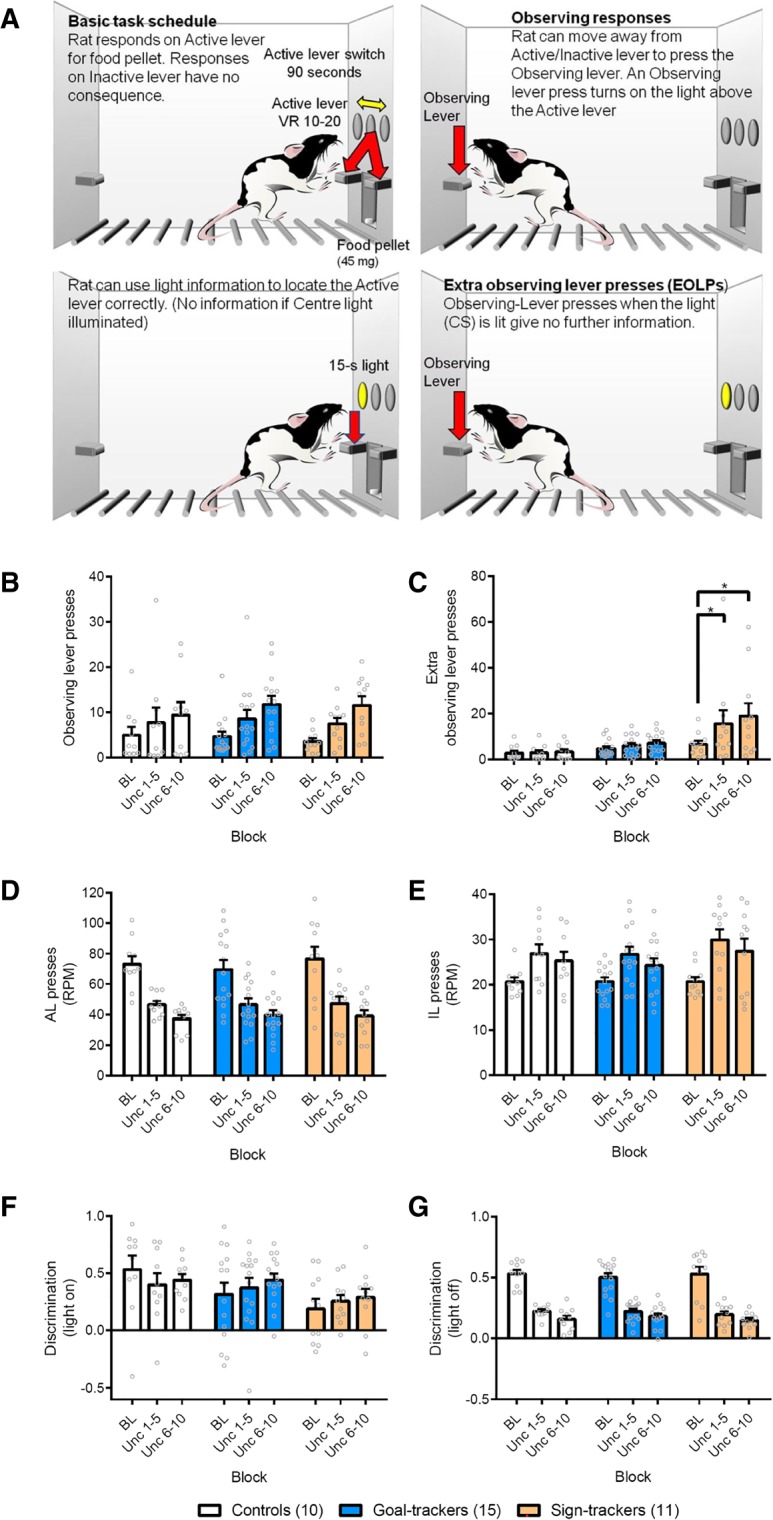
Effects of uncertainty on performance in the ORT. (*A*) Schematic of the observing response task. (*B*) Uncertainty increased functional observing lever presses (OLPs) in all animals. (*C*) Dysfunctional extra observing lever presses (eOLPs) were only increased in sign-trackers in response to uncertainty ((*) *P* < 0.05 vs. controls). (*D*) There were no differences in the rate of active lever pressing across groups, with all groups decreasing active lever pressing (i.e., becoming less accurate) during the uncertainty sessions. (*E*) Similarly, all groups increased their rate of incorrect lever pressing during the uncertainty sessions. (*F*) All groups could use the light to discriminate between the active and incorrect levers during baseline and uncertainty sessions. (*G*) Discrimination with the light off decreased in all groups during the uncertainty sessions, consistent with the task being less predictable. Data are displayed in five-session blocks across baseline (BL) and uncertainty (Unc) sessions. Values represent the mean + SEM. RPM: rate per minute. Group sizes: controls, *n* = 10; goal-trackers, *n* = 15; sign-trackers, *n* = 11. Figure 1A adapted from d'Angelo et al. (2017) and reproduced under CC BY, http://creativecommons.org/licenses/by/4.0/.

Considering the adaptive and maladaptive nature of functional and dysfunctional checking, respectively, we hypothesized that these two behaviors may be supported by different psychological mechanisms. Increases in checking correlate with uncertainty and anxiety as described above, consistent with dysfunction of the security motivation system, but we propose that the security motivation system specifically contributes to functional checking behavior. In contrast, the compulsive and dysfunctional checking that is specific to OCD, and not generalized anxiety or depression, we hypothesize to reflect uncontrolled habitual responding supported by the appetitive motivational system (Robbins et al. 2012). This hypothesis would predict that functional and dysfunctional checking would be differentially modulated by manipulations targeting the security motivation and appetitive motivational systems, respectively.

We tested the relationship between the security motivation system and checking using three different approaches. First, we manipulated the level of uncertainty in task reinforcement, as in our previous research (Eagle et al. 2014; d'Angelo et al. 2017; Eagle et al. submitted), and second, we modulated levels of perceived threat on the ORT by presenting threat-associated contextual cues that animals had experienced separately during Pavlovian fear conditioning. Third, we modified the task to assess the impact of actual (rather than perceived) threat on checking by punishing incorrect responses with a mild electric footshock on a new version of the task, the aversive ORT (aORT). We predicted that all of these anxiogenic manipulations would increase functional checking.

To investigate the relationship between dysfunctional checking and the appetitive motivational system, we capitalized on previous research describing individual differences in responses to appetitive environmental cues. It is well-established that animals show different conditioned responses to environmental Pavlovian cues predictive of reward, either approaching the location of reward delivery (“goal-tracking”) or the cue itself (“sign-tracking”). These different behavioral responses to a Pavlovian cue are considered to reflect the extent to which the cue acts as an incentive stimulus (Robinson and Flagel 2009), and are associated with alterations in mesolimbic dopamine signaling (Flagel et al. 2007, 2011; Lopez et al. 2015; Fraser et al. 2016). Based on evidence that sign-trackers show an increased propensity for maladaptive behavior including action impulsivity (Lovic et al. 2011; King et al. 2016), increased behavioral disinhibition and greater sensitivity to environmental cues (Flagel et al. 2010), we speculated that sign-trackers may show a reduced ability to control their dysfunctional checking on the ORT. We observed in a separate study (Eagle et al., submitted) that animals classified as sign-trackers based on prior autoshaping subsequently show elevated levels of dysfunctional checking on the ORT. Here we sought to replicate and extend this finding, by also investigating differences in the aversive motivational system of sign-trackers and goal-trackers.

Thus, the present study investigated the psychological mechanisms underlying functional and dysfunctional checking behavior in a fully translational, rodent-to-human analog of checking in OCD. We hypothesized that functional and dysfunctional checking behaviors are supported, respectively, by aversive (security motivation) and appetitive representations within the motivational system. We specifically predicted that: (i) presentation of anxiogenic cues, whether uncertainty, perceived threat (threat-associated contextual cues) or actual threat (punishment of incorrect responding), would increase functional, but not dysfunctional checking responses; and (ii) that sign-trackers would be selectively more sensitive to appetitive Pavlovian cues, manifest as increased dysfunctional checking independent of all experimental manipulations.

## Results

### Experiment 1: effects of uncertainty and anxiogenic stimuli on checking in goal- and sign-trackers

#### Classification of animals into sign-tracking, goal-tracking, and intermediate groups

Animals were trained to associate the to-be-observing lever with sucrose pellet delivery in a food magazine located on the opposite side of the conditioning chamber through a Pavlovian conditioned approach (autoshaping) procedure. Thirty-seven animals underwent Pavlovian conditioning, while 10 animals served as unpaired controls that received the same number and frequency of lever presentations but received all sucrose pellets at the start of the session. All animals that underwent Pavlovian conditioning were subsequently classified as sign-trackers, goal-trackers, or intermediate phenotypes depending on whether approach was toward the lever, the magazine or both, respectively. Following classification (see Materials and Methods for details), the cohort consisted of 11 sign-trackers, 11 intermediates, and 15 goal-trackers. Intermediate animals were not included in subsequent analyses. Consistent with these group assignments, goal-trackers made greater numbers of magazine entries during the autoshaping sessions [Supplemental Fig. 1A; Group: *F*_(2,33)_ = 17.3, *P* < 0.0001, η^2^ = 0.51; Group × Session: *F*_(5.4,88.6)_ = 2.31, *P* = 0.047, η^2^ = 0.12; Šidák-corrected pairwise comparisons showed that goal-trackers made more magazine entries than controls in all sessions, all *P*’s < 0.02]. Sign-trackers approached the lever more than the other experimental groups throughout training [Supplemental Fig. 1B; Group: *F*_(2,33)_ = 7.63, *P* = 0.002, η^2^ = 0.32; Group × Session: *F*_(7.4,122.6)_ = 1.31, *P* = 0.25; Šidák-corrected pairwise comparisons showed that sign-trackers made more lever approaches than both goal-trackers, *P* = 0.002, and controls, *P* = 0.024].

#### Checking was increased by uncertainty, with dysfunctional checking being exacerbated in sign-trackers

Animals were trained on the full “observing response task” (ORT) and once responding was stable, their performance under conditions of increased uncertainty was assessed. Consistent with our predictions, all animals increased the numbers of functional observing lever presses (OLPs) made under conditions of uncertainty, regardless of phenotype [Fig. 1B; Block: *F*_(1.8,58.5)_ = 29.5, *P* < 0.0001, η^2^ = 0.47; Group: *F* < 1; Block × Group: *F* < 1]. All animals also made greater numbers of dysfunctional extra observing lever presses [eOLPs; Fig. 1C; Block: *F*_(1.5,48.7)_ = 4.92, *P* = 0.02, η^2^ = 0.13; Block × Group: *F*_(3.0,48.7)_ = 2.61, *P* = 0.062, η^2^ = 0.14]. Testing our a priori hypothesis that sign-trackers would show more dysfunctional checking, we found that sign-trackers made more eOLPs than both goal-trackers [*P* = 0.04, *d* = 0.84] and controls [*P* = 0.008, *d* = 1.17]. Thus, uncertainty increased both functional and dysfunctional checking in all animals, but consistent with findings in OCD patients (Tolin et al. 2003), sign-trackers showed exacerbated dysfunctional checking compared to the other groups.

Uncertainty affected other measures of responding in the ORT as expected, based on our previous data (Eagle et al. 2014; d'Angelo et al. 2017). Under conditions of uncertainty, overall rates of active (Fig. 1D) and incorrect (Fig. 1E) lever pressing decreased, though equally for all groups [Block: *F*_(1.2,38.4)_ = 143.1, *P* < 0.0001, η^2^ = 0.81; Group: *F* < 1; Group × Block: *F* < 1]. Uncertainty did not impair the ability of the animals to use the cue light to guide responding [Fig. 1F; Block: *F* < 1; Group: *F*_(2,33)_ = 1.95, *P* = 0.16; Group × Block: *F*_(3.2,53.5)_ = 1.26, *P* = 0.30] but it did make it harder for all animals to discriminate between the active and incorrect levers while the cue light was off [Fig. 1G; Block: *F*_(1.5,47.9)_ = 179.9, *P* < 0.0001, η^2^ = 0.88; Group: *F* < 1; Group × Block: *F* < 1]. Thus, as expected, the introduction of uncertainty to the ORT led to a greater reliance on checking behavior and the consequent cue light to guide responding, as compared to the more predictable schedule in the “Baseline” sessions.

#### Threat-associated contextual cues selectively increased functional checking in goal-trackers

**Figure 2.**
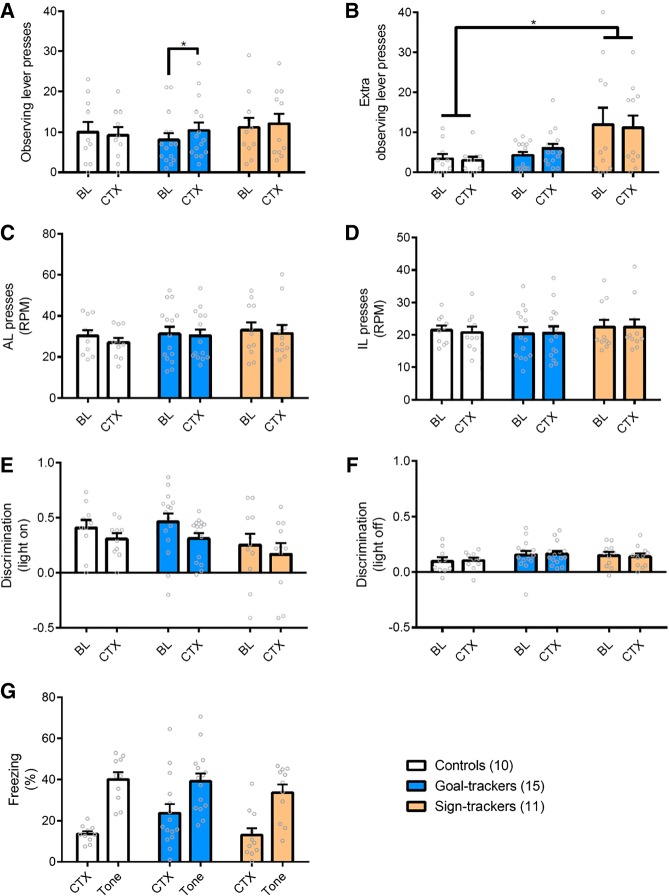
Effects of perceived threat on ORT performance. Animals were tested in the neutral (BL) context the day before fear conditioning. The next day, elements of the shock-paired context (CTX) were transferred to the boxes used for ORT. (*A*) Goal-trackers made more functional OLPs in the shock-associated context. (*) *P* < 0.05. (*B*) The shock-associated context did not affect the number of dysfunctional eOLPs made, but sign-trackers continued to show elevated dysfunctional checking related to the other groups. (*) *P* < 0.05. (*C*) There were no differences in the rate of active lever pressing between groups or across contexts. (*D*) Similarly, the rate of incorrect lever pressing remained the same in the two contexts. (*E*) All groups could use the light to discriminate between the active and incorrect levers in both contexts. (*F*) Discrimination with the light off remained the same in the two contexts, indicating that the contextual cues did not change the difficulty of the task. (*G*) The effects of perceived threat on functional OLPs in goal-trackers was not due to any differences in fear learning. When tested in the fear conditioning context, conditioned freezing was similar across all groups to both contextual (CTX) and discrete (Tone) cues. Values represent the mean + SEM. RPM: rate per minute. Group sizes: controls, *n* = 10; goal-trackers, *n* = 15; sign-trackers, *n* = 11.

To assess the impact of perceived threat on checking behavior, animals underwent Pavlovian fear conditioning to associate an electric footshock with a discrete auditory stimulus and portable contextual cues, in different chambers to those used for the ORT. All animals acquired the Pavlovian fear memory equally well, showing greater fear to the discrete tone cue than to the contextual cues [Fig. 2G; Stimulus: *F*_(1,33)_ = 40.0, *P* < 0.0001, η^2^ = 0.55; Stimulus × Group: *F* < 1; Group: *F*_(2,33)_ = 2.62, *P* = 0.09]. However, despite producing equally strong fear memories, presentation of threat-associated contextual cues did not affect responding on the ORT in the same way for goal-trackers and sign-trackers. Only goal-trackers increased functional OLPs in the presence of threat-associated contextual cues [Fig. 2A; Context: *F*_(1,33)_ = 2.44, *P* = 0.13; Group: *F* < 1; Context × Group: *F*_(2,33)_ = 3.09, *P* = .06, η^2^ = 0.16; comparing responding in the presence and absence of threat-associated contextual cues, only goal-trackers increased responding, *P* = 0.018, *d* = 0.80, all other groups *P* > 0.72]. In contrast, threat-associated contextual cues did not alter levels of dysfunctional eOLPs [Fig. 2B; Context: *F* < 1; Context × Group: *F* < 1], although sign-trackers continued to make greater numbers of eOLPs throughout testing as compared to controls [Group: *F*_(2,33)_ = 4.41, *P* = 0.02, η^2^ = 0.21; post-hoc: *P* = 0.027, *d* = 1.00]. Therefore, threat-associated contextual cues did increase checking behavior, but only functional (not dysfunctional) checking, and only in goal-trackers.

Consistent with a generalized suppression of behavior, threat-associated contextual cues produced a slight trend toward a reduced rate of active lever pressing [Fig. 2C; Context: *F*_(1,33)_ = 3.82, *P* = 0.06, η^2^ = 0.10; Group: *F* < 1; Group × Context: *F* < 1] though the low rate of incorrect lever pressing was unaffected [Fig. 2D; Context: *F* < 1; Group: *F* < 1; Group × Context: *F* < 1]. The ability of animals to use the light CS to guide lever choice was also reduced in the presence of threat-associated contextual cues [Fig. 2E; Context: *F*_(1,33)_ = 6.05, *P* = 0.019, η^2^ = 0.16; Context × Group: *F* < 1], though lever choice with the light off was not altered in either the threat-associated and neutral contexts [Fig. 2F; Context: *F* < 1; Context × Group: *F* < 1].

Overall, Experiment 1 showed that while uncertainty increased functional checking in all animals, only sign-trackers showed elevated levels of dysfunctional checking. Furthermore, perceived threat (by presentation of threat-associated contextual cues) selectively increased functional checking in goal-trackers only.

### Experiment 2: effects of punishing incorrect responses in the ORT

Experiment 1 assessed the impact of anxiogenic stimuli on checking through manipulations of uncertainty and in the presence of threat-associated cues. Experiment 2 tested the impact of aversive outcomes on checking behavior, by using the “aversive ORT” (aORT), in which responding on the incorrect lever was punished directly with an electric footshock. The intensity of this foot shock was increased across sessions from 0.1 to 0.5 mA in increments of 0.1 mA every two sessions. The shocks were then disabled before the effect of separately shock-paired contextual stimuli on checking was determined.

#### Classification of animals into sign-tracking, goal-tracking, and intermediate groups

Animals that had undergone autoshaping were classified on their Pavlovian conditioned approach behavior as for Experiment 1. As expected, animals classified as goal-trackers made more magazine entries [Supplemental Fig. 1C; Group: *F*_(2,30)_ = 67.5, *P* < 0.001, η^2^ = 0.53; Group × Session: *F*_(8.2,123.0)_ = 3.97, *P* < 0.001, η^2^ = 0.21: Session: *F*_(4.1,123.0)_ = 2.69, *P* = 0.03, η^2^ = 0.08] and those classified as sign-trackers made more approaches toward the lever [Supplemental Fig. 1D; Group: *F*_(2,30)_ = 67.5, *P* < 0.001, η^2^ = 0.82; Session: *F*_(1.8,53.9)_ = 21.6, *P* < 0.001, η^2^ = 0.42; Session × Group: *F*_(3.6,53.9)_ = 18.5, *P* < 0.001, η^2^ = 0.55]. This cohort consisted of six sign-trackers, 15 intermediates, and 17 goal-trackers.

#### Punishment of incorrect responding increased both functional and dysfunctional checking, and persistently so in sign-trackers

**Figure 3.**
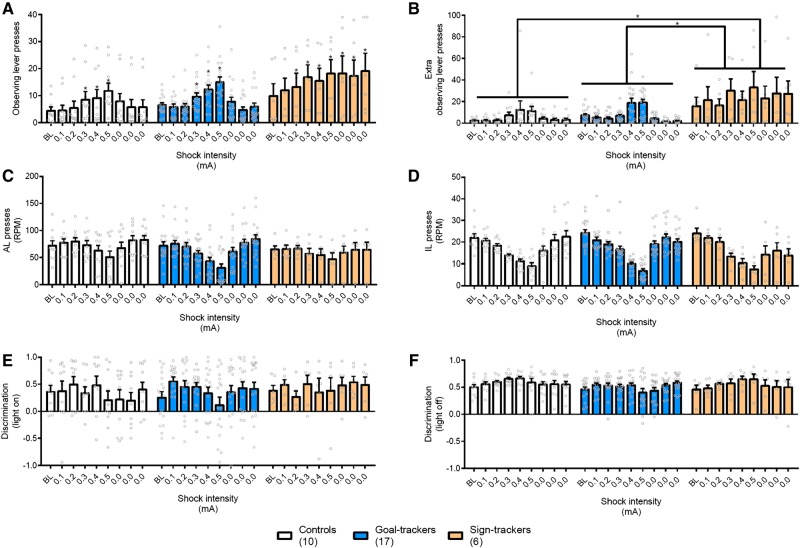
Effects of actual threat on ORT performance. (*A*) The introduction of punishment led to an increase in functional OLPs in all groups at shock magnitudes of 0.3 mA and above. Once the shocks were disabled, goal-trackers and controls returned to baseline levels of OLPs, while sign-trackers continued to show elevated checking. (*B*) Dysfunctional eOLPs increased in all groups at shock magnitudes of 0.4 mA and above but returned to baseline once shocks were no longer delivered. Note that sign-trackers showed an elevated baseline compared to the other groups. (*C*) Actual threat reduced the rate of active lever pressing at shock magnitudes of 0.4 mA and above but returned to baseline once shocks were no longer delivered. (*D*) The rate of incorrect lever presses decreased from baseline at shock magnitudes of 0.2 mA and above, consistent with the shock punishing this behavior. The rate of incorrect responses returned to baseline after the punishment was removed. (*E*) All groups could use the light to discriminate between the active and incorrect levers in all sessions. (*F*) Discrimination with the light off was improved at the 0.4 mA intensity but did not differ from baseline. Data are displayed as two-session blocks across preshock baseline (BL) and individual shock values, including subsequent unpunished sessions. Values represent the mean + SEM. RPM: rate per minute. Group sizes: controls, *n* = 10; goal-trackers, *n* = 17; sign-trackers, *n* = 6.

The introduction of punishment to the ORT increased functional checking in all animals in a shock intensity-dependent fashion [Fig. 3A, Intensity: *F*_(3.9, 118.2)_ = 18.7, *P* < 0.001, η^2^ = 0.38; post-hoc tests revealed that all groups increased OLPs made in response to the 0.3, 0.4, and 0.5 mA shock intensities, all *P's* < 0.03]. However, when incorrect responding was no longer punished, the functional OLP checking responses of sign-trackers were impervious to the removal of the shock [Intensity × Group: *F*_(7.8,118.2)_ = 3.66, *P* = 0.001, η^2^ = 0.20] and did not return to baseline like goal-trackers and controls [*P* < 0.0001, *d* = 1.25; *P* = 0.0005, *d* = 1.34; *P* < 0.0001, *d* = 1.67, for the three respective blocks of extinction tests]. Thus, while functional checking increased in all groups in an adaptive manner in response to the introduction of punishment to the task, sign-trackers were unable to adaptively reduce their functional checking when punishment was no longer applied.

Dysfunctional checking (eOLPs; Fig. 3B) was also affected by shock intensity, but differently to functional checking. As in Experiment 1, sign-trackers showed more dysfunctional checking throughout testing, regardless of the punishment contingency [Group: *F*_(2,30)_ = 5.89, *P* = 0.007, η^2^ = 0.28; post-hoc tests showed greater numbers of eOLPs than both goal trackers at *P* = 0.013, *d* = 0.90 and controls at *P* = 0.010, *d* = 1.02]. However, with the introduction of punishment, all groups increased their dysfunctional checking in a shock intensity-dependent manner [Intensity: *F*_(2.7,80.3)_ = 6.56, *P* = 0.001, η^2^ = 0.18; Intensity × Group: *F*_(5.4,80.3)_ = 1.81, *P* = 0.12; eOLPs were increased at 0.4 and 0.5 mA intensities, *P* = 0.0001, *d* = 0.47, and *P* < 0.0001, *d* = 0.67, respectively]. Dysfunctional checking returned to baseline levels when incorrect responding was no longer punished in all groups [*P* > 0.11, *d* < 0.13], though sign-trackers continued to show greater levels of dysfunctional checking than the other groups. Therefore, as for Experiment 1, sign-trackers showed higher levels of dysfunctional checking overall, but punishment led to increases in dysfunctional checking in all animals.

As for the presentation of anxiogenic stimuli in Experiment 1, presentation of aversive stimuli—in this case, punishment of incorrect responding—suppressed overall rates of responding in all animals, in a shock intensity-dependent manner [Fig. 3C; Intensity: *F*_(3.2,95.7)_ = 14.0, *P* < 0.001, η^2^ = 0.32; Group: *F* < 1; Group × Intensity: *F*_(6.4,95.7)_ = 1.56, *P* = 0.16; post-hoc tests revealed that the rate of active lever pressing was decreased at 0.4 mA, *P* < 0.0001, *d* = 0.58, and 0.5 mA, *P* < 0.0001, *d* = 0.97]. Once shocks were no longer delivered in the ORT sessions, no suppression was observed and the rates of active lever presses returned to baseline levels [all *P*’s > 0.11, *d* < 0.51]. As expected, punishment of incorrect responding also decreased the rate of incorrect lever pressing, and at lower shock intensities than for active lever pressing [Fig. 3D; Intensity: *F*_(4.4,130.1)_ = 27.8, *P* < 0.0001, η^2^ = 0.48; Group: *F* < 1; Group × Intensity: *F*_(8.7,130.1)_ = 1.4., *P* = 0.19; post-hoc tests showed that the rate of incorrect lever presses was decreased at the 0.2–0.5 mA shock intensities, *P* < 0.002, *d* > 0.87]. This reduction in incorrect lever pressing also affected discrimination measures, as animals better discriminated between the two levers in the shocked sessions when the light was off [Fig. 3F; Intensity: *F*_(4.0,120.8)_ = 2.48, *P* = 0.047, η^2^ = 0.08; Group: *F* < 1; Intensity × Group: *F*_(8.1,120.8)_ = 1.59, *P* = 0.14], though lever choice when the cue light was on did not differ between the shocked and nonshocked sessions [Fig. 3E; Intensity: *F*_(5.3,159.3)_ = 1.12, *P* = 0.35; Group: *F* < 1; Intensity × Group: *F* < 1]. This suggests, expectedly, that rats avoided pressing the incorrect lever after this response was punished.

#### Threat-associated contextual cues selectively increased functional checking in goal-trackers, even in animals with experience of the aORT

**Figure 4.**
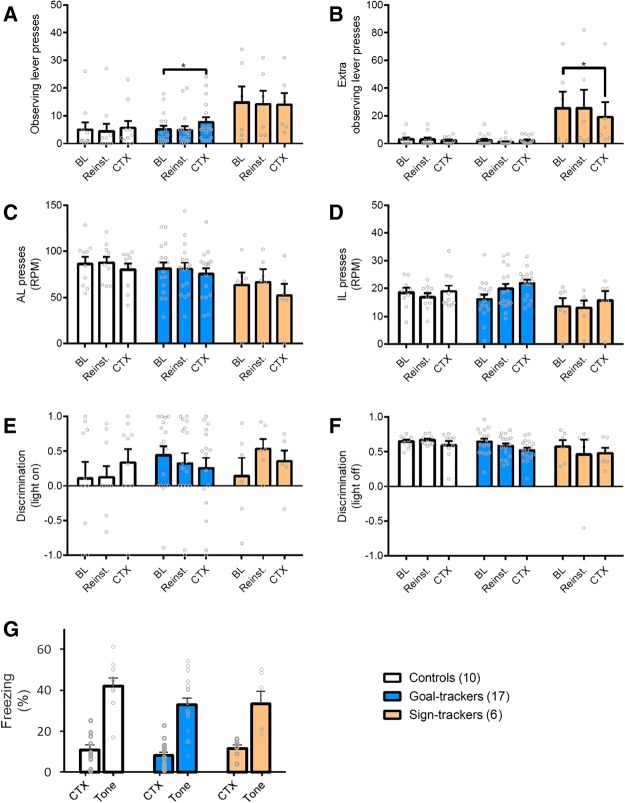
Effects of perceived threat on ORT performance, following experience of the aversive ORT. Animals were tested in the neutral context the day before fear conditioning (BL). In order to determine whether the shock alone could reinstate behavior observed during testing in the aORT animals were tested in the neutral context the next day (Reinst.), before being tested in the shock-paired context the day after this (CTX). (*A*) Goal-trackers made more functional OLPs in the shock-associated context. (*) *P* < 0.05. (*B*) Sign-trackers continued to show elevated levels of dysfunctional eOLPs compared to the other groups, but eOLPs were reduced by perceived threat following previous experience of punishment. (*) *P* < 0.05. (*C*) There were no differences in the rate of active lever pressing between groups or across contexts. (*D*) Similarly, the rate of incorrect lever pressing remained the same across all contexts. (*E*) All groups could use the light to discriminate between the active and incorrect levers across contexts. (*F*) Discrimination with the light off was the same across the contexts, indicating that perceived threat did not change the difficulty of the task. (*G*) The effects of perceived threat on checking in the ORT were not due to any differences in fear learning. When tested in the fear conditioning context, conditioned freezing was similar across all groups to both contextual (CTX) and discrete (Tone) cues. Values represent the mean + SEM. Group sizes: controls, *n* = 10; goal-trackers, *n* = 17; sign-trackers, *n* = 6.

Following completion of testing on the aORT, we sought to determine whether prior experience of punishment would affect the capacity of anxiogenic stimuli to alter functional and dysfunctional checking in sign-trackers and goal-trackers. Specifically, we wanted to test whether subsequent shock exposure in the contextual fear conditioning (CFC) procedure would reinstate the levels of functional checking previously observed during the aORT sessions. As for Experiment 1, rats underwent Pavlovian CFC in a separate chamber, containing stimuli that could be transferred to the ORT chambers. To determine whether the footshock delivered during CFC would reinstate the responding observed on the aORT, animals were first tested in the normal ORT chambers 24 h after CFC in a “reinstatement” test, and 48 h after conditioning, they were tested in the ORT chambers in the presence of the threat-associated contextual cues. All of the animals showed similar levels of conditioned fear when tested in the fear conditioning context and presented with the discrete auditory cue associated with shock, with fear to the cue being higher than to the context in all animals [Fig. 4G; Stimulus: *F*_(1,30)_ = 102.5, *P* < 0.0001, η^2^ = 0.77; Stimulus × Group: *F*_(2,30)_ = 1.07, *P* = 0.36; Group: *F*_(2,30)_ = 1.65, *P* = 0.21].

Prior experience of the aORT did not alter the effects on checking of threat-associated contextual cues that were previously observed in Experiment 1. Again, only goal-trackers elevated their functional checking when tested in the presence of threat-associated contextual cues [Fig. 4A; Context: *F*_(1.3,39.8)_ = 2.26, *P* = 0.13; Context × Group: *F*_(2.7,39.8)_ = 1.59, *P* = 0.21; based on our a priori hypothesis and the results of Experiment 1, pairwise comparisons revealed that goal-trackers, *P* = 0.01, *d* = 0.79, but no other group, all *P*’s > 0.99, increased their functional checking in the presence of threat-associated contextual cues]. Furthermore, the delivery of footshock in the fear conditioning session did not reinstate the responding observed previous on the aORT, with no observed differences in functional checking in the “Reinstatement” test [Fig. 4A, controls: *P* = 0.99, *d* = 0.51; sign-trackers: *P* = 0.99, *d* = 0.29; goal-trackers: *P* > 0.99, *d* = 0.15]. The patterns of dysfunctional checking (Fig. 4B) also replicated those observed in Experiment 1. Sign-trackers showed higher levels of dysfunctional checking [Group: *F*_(2,30)_ = 7.67, *P* = 0.002, η^2^ = 0.34; post-hoc tests showed that sign-trackers made more eOLPs than both controls at *P* = 0.007, *d* = 1.01, and goal-trackers at *P* = 0.002, *d* = 1.06], though in contrast to Experiment 1, these decreased in the presence of threat-associated contextual cues [Context: *F*_(1.8,55.2)_ = 2.99, *P* = 0.06; Context × Group: *F*_(3.7,55.2)_ = 2.48, *P* = 0.06; post-hoc tests showed that sign-trackers, *P* = 0.01, *d* = 0.48, but not the other groups, all *P*’s > 0.87, decreased dysfunctional checking in this context]. Dysfunctional checking in the “Reinstatement” test was no different than previous baseline measurements [all *P*’s > 0.67, *d* < 0.30] indicating that the experience of shock in CFC did not reinstate levels of responding to those observed on the shocked aORT sessions.

Further replicating the findings of Experiment 1, threat-associated contextual cues suppressed the rate of active lever pressing [Fig. 4C; Context: *F*_(1.6,47.4)_ = 12.4, *P* < 0.001, η^2^ = 0.29; Group: *F*_(2,30)_ = 1.69, *P* = 0.20; Context × Group: *F* < 1], while the rate of incorrect lever pressing remained unaffected [Fig. 4D; Context: *F*_(1.8,53.3)_ = 2.71, *P* = 0.08; Group: *F*_(2,30)_ = 2.29, *P* = 0.12; Context × Group: *F*_(3.6,53.3)_ = 1.48, *P* = 0.23]. This time, discrimination between the active and incorrect levers was not affected by the presence of threat-associated contextual cues, either when the cue light was on [Fig. 4E; Context: *F* < 1; Group: *F* < 1; Context × Group: *F*_(4.0,59.6)_ = 1.68, *P* = 0.17] or off [Fig. 4F; Context: *F*_(1.8,53.8)_ = 2.72, *P* = 0.08; Group: *F*_(2,30)_ = 1.23, *P* = 0.31; Context × Group: *F* < 1].

Overall, Experiment 2 showed that actual threat (punishment of incorrect responses with an electric footshock) increased functional checking in all animals. Dysfunctional checking was also increased in all animals, though it remained elevated in sign-trackers compared to goal-trackers. Importantly, while goal-trackers were able to adaptively reduce their levels of checking when incorrect responses were no longer punished, sign-trackers continued to show elevated responding, indicating that they were insensitive to the changes in reinforcement contingencies.

## Discussion

Excessive checking behavior is common in OCD, but checking itself is a normal part of the behavioral repertoire. We have previously argued (Eagle et al. 2014) that the distinction between functional and dysfunctional checking is key to understanding OCD, and here we sought to examine the psychological bases of these two types of checking behavior. Specifically, we aimed to determine the impact of individual differences in sensitivity to the incentive salience of appetitive Pavlovian cues, presentation of threat-associated contextual cues, and direct punishment of incorrect responses on functional and dysfunctional checking in the ORT. We hypothesized that functional checking would primarily be driven by the aversive motivational system (consistent with the dysfunctional security motivation view of Szechtman and Woody 2004). Our hypothesis that levels of dysfunctional checking would be associated with individual differences in the appetitive motivational system led us to classify animals as sign-trackers or goal-trackers prior to experimental manipulations on the ORT, and therefore also allowed us to assess the impact of both the aversive and appetitive motivational systems—and any interaction between these—on checking behavior.

Consistent with our observations in a separate study (Eagle et al., submitted), we found that rats classified as sign-trackers following autoshaping training showed higher levels of dysfunctional checking than goal-trackers or non-autoshaped controls, and that this was particularly so under conditions of uncertainty. A similar finding, that sign-tracking itself increases when reward is uncertain, has recently been reported (Anselme and Robinson 2019). Increases in functional checking were observed in all animals under the anxiogenic conditions of increased uncertainty (Experiment 1) and the punishment of incorrect responses (Experiment 2), consistent with findings from the human version of the ORT (Morein-Zamir et al. 2018) and with the view that functional checking behavior is an adaptive, information-providing response to perceived or actual threat.

However, sign-trackers were not more sensitive to aversive Pavlovian cues; the levels of dysfunctional checking were similar in both the neutral and aversive threat-associated contexts, although it should be noted that sign-trackers showed elevated levels of dysfunctional checking in the neutral context, perhaps masking any further elevation of dysfunctional checking by the presentation of threat-associated contextual cues. In contrast, goal-trackers showed an increase in their functional checking in the presence of these aversive cues, supporting the hypothesis that functional checking depends upon aversive motivational systems, but dysfunctional checking does not. Notably, these data could not be attributed to any differences in shock sensitivity or the strength of fear conditioning, as sign-trackers, goal-trackers, and controls all showed equivalent fear memory when explicitly tested on contextual and discrete Pavlovian fear conditioning. Furthermore, when incorrect responses were explicitly punished in the aversive ORT (aORT) all groups increased their functional checking behavior, as predicted. However, when incorrect lever presses were no longer punished, only sign-trackers continued to check at high levels; other groups’ checking responses returned to baseline levels. These data support the view that functional and dysfunctional checking depend differentially on the aversive and appetitive motivational systems, respectively.

While sign-trackers’ functional checking responses showed enduring changes in response to punishment of incorrect responses with shock, it was the functional checking responses of goal-trackers that were sensitive to the presence of threat-associated contextual cues. Previous studies have shown that goal-trackers freeze more to contextual cues and sign-trackers to discrete cues following Pavlovian fear conditioning (Morrow et al. 2011); however, we did not observe these differences with our more moderate fear conditioning procedure, which was attempting to modulate anxiety rather than fear per se. The levels of conditioned freezing and (critically for our purposes of assessing changes in behavior on the ORT) conditioned suppression were relatively low. Predatory Imminence Theory (Perusini and Fanselow 2015) suggests that distal threats, which are likely anxiety-mediated, can increase vigilance and risk assessment. The weaker fear responses observed to the shock-paired context in this study suggest the perceived sense of threat was more likely to induce anxiety than fear and would likely have triggered preencounter reactions. It is reasonable to hypothesize that increased vigilance and risk assessment would manifest on the ORT as increased information-seeking in the presence of shock-paired stimuli, in this case increased functional checking. However, it is not clear whether goal-trackers are particularly affected due to increased anxiety elicited by threat-associated contextual cues (Morrow et al. 2011) or due to a more generalized influence of contextual cues on instrumental behavior in this group. Goal-trackers are more susceptible to the influence of appetitive contextual cues on cocaine-seeking behavior (Saunders et al. 2014; Pitchers et al. 2017) which may suggest the latter; however, this does not account for the selective increase in functional, but not dysfunctional, checking in this group, perhaps supporting an anxiety-based account. Alternatively, all groups may be similarly susceptible to the anxiety-provoking ability of threat-associated contextual cues, but only goal-trackers respond to this anxiety by increasing their functional checking. The use of anxiogenic compounds, rather than associative cues to increase anxiety, may help in addressing this issue in future studies.

While all groups responded similarly in the aORT to the escalating intensity of punished incorrect responses by increasing their functional OLPs, once the shocks were no longer delivered, goal-trackers and control rats rapidly returned to baseline levels of functional checking. Sign-trackers, in contrast, appeared impervious to the absence of shock, continuing to check at high levels. There are at least two potential explanations for this checking behavior: one based on the informativeness of the cue produced by the checking response and the other on its reinforcing properties. The presentation of the cue produced by functional checking provides information to the animal, by indicating which lever is currently reinforced, thereby reducing uncertainty. The “uncertainty reduction hypothesis” (Berlyne 1960) posits that behaviors that result in the delivery of discriminative stimuli enable the animal to modify its behavior to be maximally advantageous in its environment. The increase in functional checking in response to uncertainty and punished incorrect lever pressing is consistent with this information-seeking theory. However, sign-trackers continued to show high levels of functional checking in the aORT, even once incorrect responses were no longer punished, in contrast to goal-trackers and control animals, who adapted their behavior in response to the altered shock contingency. This could reflect a more rapid transition to habitual responding in sign-trackers, consistent with computational models suggesting that sign-trackers place rely more heavily on the model-free (habitual) than the model-based (goal-directed) system (Lesaint et al. 2015).

An alternative view to account for the persistent functional checking in sign-trackers is the “conditioned reinforcement hypothesis” (Dinsmoor 1983). This view would suggest that functional observing responses are maintained by the capacity of the discriminative cue light to act as a conditioned reinforcer (d'Angelo et al. 2017). It is well-known that conditioned reinforcement is extremely persistent and resistant to extinction (Di Ciano and Everitt 2004) and it is thought to be one of the more pernicious ways in which cues can influence instrumental behavior in other mental health disorders, including drug addiction (Milton and Everitt 2010). Furthermore, sign-trackers more readily acquire behaviors dependent upon conditioned reinforcers (Yager and Robinson 2010, 2013) and in humans, there is a correlation between the extent of compulsive behavior self-reported on questionnaire measures and the capacity of reward-related cues to capture attention (Albertella et al. 2019). It remains to be tested whether greater attention to reward-related cues correlates with increased checking on the human version of the ORT (Morein-Zamir et al. 2018). However, against the conditioned reinforcement view is the finding that lesions of the nucleus accumbens core, a structure known to be critical for the expression of conditioned reinforcement (Taylor and Robbins 1986; Di Ciano et al. 2008) lead to increased functional checking on the ORT (d'Angelo et al. 2017).

Regardless of whether functional checking is supported by the informational or reinforcing value of the cue, the high levels of dysfunctional checking in sign-trackers cannot be supported by the cue itself because extra observing lever presses (eOLPs) do not result in illumination of the cue light. These responses may have been due to continued sign-tracking to the lever, presentation of which had been reinforced during autoshaping training. However, this seems unlikely, given that these animals increased their eOLPs as training in the ORT progressed, during which time the association between the observing lever presentation and reward delivery was no longer reinforced. To fully discount this possibility, future studies will classify animals as sign-trackers and goal-trackers following autoshaping in an entirely distinct apparatus, such as a touchscreen chamber.

A second, and we suggest more likely, possibility is that the operant response of checking itself became imbued with motivational significance—the incentive motivational properties once directed toward the Pavlovian cue was now directed to the observing response, akin to the “incentive habits” view of addiction (Belin et al. 2013). This would account for the high levels of functional checking in sign-trackers during extinction of the aORT, consistent with previous reports that sign-trackers are less cognitively flexible under changing reinforcement contingencies (Ahrens et al. 2016). This would also account for the high levels of dysfunctional checking that these animals show under uncertainty conditions in the standard ORT. The development of a checking habit for animals that have previously attached high incentive value to the observing lever would be sufficient to support responding on this lever, even if it is not required to illuminate the stimulus light or avoid shock. In order to test whether the checking response is habitual and compulsive, future studies will need to alter the contingency between the checking lever and the cue light, or directly punish the checking response itself, to determine whether sign-trackers selectively are insensitive to contingency degradation and counterconditioning of this response.

Overall, these data support the view that functional and dysfunctional checking are dissociable and supported by aversive and appetitive motivational processes, respectively. Functional checking behavior can be modulated by perceived threat and uncertainty, and by the capacity of discriminative environmental cues to act as conditioned reinforcers. However, dysfunctional checking appears to recruit appetitive motivational processes, likely akin to the “incentive habits” that contribute to drug-seeking behavior in drug addiction. This view of dysfunctional checking is consistent with patient data reporting increased habit learning in OCD (Gillan and Robbins 2014; Gillan et al. 2017). Individual differences in the attribution of incentive salience to environmental cues interacts with the processes underlying functional and dysfunctional checking. Ultimately, these data support a view of OCD that suggests that maladaptive emotional memories—both Pavlovian and instrumental—contribute to the development of dysfunctional behavior, thereby providing future potential targets for treatment interventions, such as the disruption of these memories by reconsolidation blockade.

## Materials and Methods

### Subjects

Subjects were 96 male Lister Hooded rats (Charles River, Bicester, UK) with weights at the start of procedures of 156–222 g for Experiment 1 (mean: 196 g) and 274–352 g for Experiment 2 (mean: 306 g). Animals were housed in groups of 4 with a cardboard tube as enrichment under a reversed light–dark cycle (lights on at 19:00). Testing took place 5 d a week, typically between 08:00 and 13:00. Before any testing began animals were food restricted and fed 10–20 g of standard laboratory chow (SDS) at the end of each day's testing, in addition to any food earned during operant sessions. One rat was excluded after being observed having multiple seizures; data from this animal have been removed from all analyses. Experiments were run as two separate squads of 48 rats. This research has been regulated under the Animals (Scientific Procedures) Act 1986 Amendment Regulations 2012 following ethical review and approval by the University of Cambridge Animal Welfare and Ethical Review Body (AWERB) and was conducted under PPL 70/7548.

### Apparatus

Rats were trained in 18 operant-conditioning chambers (Med-Associates) equipped with three levers, two of which flanked a food receptacle where 45 mg sucrose pellets (AIN-76A, Sandown Scientific) were delivered. Above of each of these levers was a stimulus light. A third lever was located on the opposite wall, above which was a white house light that remained illuminated throughout the session.

### Behavioral procedures

#### Experiment 1

In brief, animals were trained on the ORT and underwent separate autoshaping training sessions to enable classification into goal-tracking and sign-tracking phenotypes. Animals were then tested in the ORT under conditions of uncertainty, where it was more difficult for animals to discriminate between active and inactive/incorrect levers without the use of the discriminative stimulus, which could be illuminated by responding on the observing lever. In the “standard” version of the ORT used in Experiment 1, the incorrect lever was not reinforced and can therefore be considered equivalent to “inactive” lever pressing in our previous work. However, in the aversive ORT used in Experiment 2, pressing the incorrect lever was punished and it is therefore inappropriate to refer to these as “inactive” lever presses. For consistency, we have referred to these as “incorrect” lever presses in both experiments reported here.

Following this, the effects of threat-associated stimulus were assessed by fear conditioning animals to portable contextual and discrete stimuli. The effect of presenting these threat-associated stimuli on performance in the ORT was subsequently examined, in addition to the effect of contextual and discrete shock-paired stimuli on conditioned freezing behavior.

##### Observing response task

Animals were trained on the ORT as previously described (Eagle et al. 2014). Animals were initially trained to discriminate between active and incorrect levers. The identity of the active and incorrect levers changed during the sessions on a fixed time (FT) 90s schedule during initial training and was signaled by illumination of a cue light located above the active lever. The observing lever remained retracted during these initial sessions. Responses on the active lever were reinforced on a progressively leaner fixed ratio (FR) and variable ratio (VR) schedules of reinforcement until all rats were responding on a VR15 schedule (range: 10–20), except where stated. These initial behavioral training sessions were conducted twice a day. When discrimination training was entering its final stages, animals underwent autoshaping sessions (Pavlovian conditioned approach training—see below) for the first session of each day, followed by discrimination training for the second session of the day.

Following reliable discrimination of the active and incorrect levers, and completion of autoshaping training, animals were trained in the full version of the ORT. The observing lever was now presented; responses on this third lever illuminated the cue lights, which were otherwise turned off. Responses on the observing lever resulted in illumination of the cue light above the active lever for 30 sec for the first four sessions, and 15 sec thereafter. The observing lever and surrounding areas were baited with powdered reward pellets for the first two sessions of ORT training to encourage exploration and engagement. Unless stated otherwise, active lever presses continued to be reinforced on a VR15 schedule and the identity of active and incorrect levers changed every 90 sec.

Responses on the ORT were collected automatically by a computer running the Whisker Control server (Cardinal and Aitken 2010). Six dependent variables were measured in the ORT sessions: (i) o*bserving lever presses (OLPs)*, which were functional observing presses that resulted in the illumination of the light about the currently active lever; (ii) e*xtra observing lever presses (eOLPs)*, which were dysfunctional presses on the observing lever, performed while the stimulus light was already illuminated. These responses did not prolong the duration of stimulus light illumination; (iii) a*ctive lever presses (rate per minute)*; (iv) *incorrect lever presses (rate per minute)*; (v) *discrimination (light on)*; and (vi) *discrimination (light off)*, which were measures of the accuracy of lever pressing with the light on and off, respectively. This was calculated as

(Activeleverpresses−Incorrectleverpresses)/Active+Incorrectleverpresses.

In cases where animals made zero responses with the light on, animals were assigned a “light on discrimination value” of zero.

##### Autoshaping

During autoshaping sessions, the lever that would subsequently be used as the observing lever was presented 30 times. Ten seconds after its presentation it was retracted, followed by delivery of a single sucrose pellet. Ten animals were assigned to a control group in which lever retraction was not paired with pellet delivery; instead, these animals received 30 pellets at the start of each session, before the house light was illuminated. Animals received a total of 17 autoshaping sessions. Animals were classified as goal-trackers, sign-trackers, or intermediates based on behavior during these sessions (see below). Intermediates were excluded from statistical analysis but continued to be tested with the other subjects. The data from these animals can be found in the Supplemental Data Files (https://doi.org/10.17863/CAM.41573).

##### Uncertainty condition of the ORT

After 10 sessions of training in the ORT animals underwent uncertainty training. During these sessions, the active and incorrect levers changed on a variable time (VT) 70 sec schedule (20–120 sec). Active lever presses were now reinforced on a variable (VI) 15 sec schedule (10–20 sec).

##### Fear conditioning

After testing under uncertainty animals underwent CFC. Procedures were adapted from Muravieva and Alberini (2010). All fear conditioning sessions took place 1–2 h following a session in the ORT. CFC sessions were conducted in different physical boxes to those for ORT sessions and were located in a different testing room. Several modifications were made to these boxes to try to ensure that they were readily distinguishable from the ORT context. The two Perspex walls had striped wallpaper affixed to them, the waste pan had striped paper placed inside, ventilation fans were turned on in the boxes, the boxes were scented with ginger odor (Ginger Organic Essential Oil, Tisserand Aromatherapy) and animals were transported in different carry boxes to those normally used. Normal testing boxes were scented with lemon (Lemon Organic Essential Oil, Tisserand Aromatherapy) during testing sessions to mask any ginger from nearby testing rooms or the previous days’ testing.

Animals were first habituated to these modified boxes for a 5-min session, where the house light was illuminated but no shocks or tones were delivered. The following day, animals were placed in the chambers for 3-min fear conditioning session. After 120 sec of habituation, a tone was presented for 30 sec which coterminated with delivery of a footshock (1.0 mA, 1 sec). The house light remained illuminated for a further 30 sec and the animal was removed. Boxes were cleaned with 70% ethanol between fear conditioning sessions.

Testing took place the following day. The effect of threat-associated contextual cues on responding in the ORT was assessed first. These sessions took place in the chambers in which animals had been trained for the ORT, now modified to include the contextual cues present for the fear conditioning session. Later, on the same day, animals underwent a contextual fear memory retention test, during which they were placed in the fear conditioning chambers for 3 min. Twenty-four hours after this test, fear to the tone was assessed by presenting four 30 sec tones, each separated by 120 sec. These sessions took place in the chambers used for fear conditioning but were modified to resemble the chambers used for ORT training (lemon scent, wallpapers removed, fans turned off, normal carry boxes).

Fear conditioning training and testing sessions were video recorded and manually scored offline by an experimenter blind to group assignment (ALM), using a custom script written for PsychToolbox for MATLAB, produced by GHV. Freezing, defined as a cessation of all movement other than those required for respiration, was scored continuously, with the values being expressed as a percentage of the total time that could be spent freezing.

#### Experiment 2

Animals in this experiment underwent pretraining and autoshaping sessions identical to those in Experiment 1. However, instead of the introduction of uncertainty to increase checking, animals underwent testing in the aORT, where incorrect lever presses were punished with a mild footshock, of increasing intensity. The shocks were then disabled for six sessions before animals underwent CFC, as described for Experiment 1.

##### Aversive ORT

In the aversive version of the ORT shocks were delivered contingently upon incorrect lever presses, on the same schedule that the active lever delivered pellets (typically VR15). Animals underwent testing at progressively increased intensities of footshock (0.1–0.5 mA, 0.5 sec shock, with shock intensity increasing in increments of 0.1 mA every two sessions). The maximum number of shocks an animal could receive in a single session was 30; sessions continued in the absence of any further shocks if this limit was reached. The average of both sessions at each intensity was used for analysis. Following completion of testing under the five different intensities in the aORT animals continued testing in the ORT in the absence of shock for a further 4 d.

##### Fear conditioning

Following aORT training, animals underwent CFC as in the previous experiment. However, in order to dissociate the possible reinstating ability of experiencing shock in a novel context (Bouton and Bolles 1979) animals were tested in the context usually used for ORT the day after CFC. Animals were tested in the ORT the following day (2 d after fear conditioning) in the presence of threat-associated contextual stimuli. Contextual and discrete fear associations were tested as described for Experiment 1.

#### Data analysis

##### Autoshaping

Grouping of animals to autoshaping and control groups was conducted based on total rate of active lever pressing and discrimination ratios; efforts were made to ensure that no pre-existing differences existed between groups before autoshaping training began. Grouping of ST, GT, and INT animals was based on the ratio of *lever presses:magazine entries during lever presentation* during the last 2 d of autoshaping training and was conducted by an experimenter blind to performance in the ORT (DME). Attempts were made to determine subgroups based on clear splits on the distribution of animals’ responding. Although more standardized protocols have been proposed for the classification of goal and sign-trackers (Meyer et al. 2012), we found that these measures produced large numbers of goal-trackers, most likely due to the high rates of magazine entries in all animals. These high numbers of magazine entries were likely the result of the animals’ ongoing instrumental training in the ORT. Intermediate animals were excluded from the analysis, but their data is available in the accompanying data set (https://doi.org/10.17863/CAM.41573).

##### ORT and aORT

Baseline performance in the ORT in Experiment 1 was based on the last five sessions of task performance. The average of two five-session blocks of uncertainty sessions were used for analysis (d'Angelo et al. 2017). Data were averaged because animals’ responding varied from day-to-day; the use of a five session-block average provides information about the progression of responding without interference from these fluctuations. Non-averaged data are provided in the accompanying data set (https://doi.org/10.17863/CAM.41573). Baseline performance for use in the aORT was obtained from the two sessions before aORT testing began. The values for each shock intensity are an average of the 2 d testing.

#### Statistical analyses

Experiments were analyzed with mixed 2 × 2 ANOVAs. The degrees of freedom for all analyses with repeated measures factors with more than two levels were adjusted with the Greenhouse-Geisser correction where Mauchly's test indicated that sphericity had been violated. Significant main effects and interactions were followed up with simple effects analysis, where the baseline sessions were compared against all other sessions, with the Šidák correction for multiple comparisons applied. Statistical analysis was conducted with GraphPad Prism (ver. 6.07) for Windows. Partial eta squared (η^2^) was calculated in SPSS (ver. 17, IBM Inc.). Cohen's d was calculated for all significant post-hoc tests. In cases where post-hoc tests report the results of within-subject comparisons *d*_z_ values are reported, (calculated as *d*_*z*_ = *m*_1_ − *m*_2_/SD_diff_) In cases of significant main effects, but no significant interaction, effect sizes were calculated for the averaged of values across the range of values of the second factor.

## Supplementary Material

Supplemental Material
