# Supplementary material for: Checking responses of goal- and sign-trackers are differentially affected by threat in a rodent analog of obsessive–compulsive disorder
Source: Learn Mem. 2020 May;27(5):190–200. doi: 10.1101/lm.050260.119 (PMC7164513; doi:10.1101/lm.050260.119)
Supplement: Supplemental Material [file supp_27_5_190__index.html]

Supplemental Material 

# Checking responses of goal- and sign-trackers are differentially affected by threat in a rodent analog of obsessive–compulsive disorder

## Supplemental Material

- Supplemental\_Figure1.pdf
- Supplemental\_Legend.docx
