# Supplementary material for: Checking responses of goal- and sign-trackers are differentially affected by threat in a rodent analog of obsessive–compulsive disorder
Source: Learn Mem. 2020 May;27(5):190–200. doi: 10.1101/lm.050260.119 (PMC7164513; doi:10.1101/lm.050260.119)
Supplement: Supplemental Material [file supp_27.5.190_Supplemental_Legend.docx]

**Supplementary Figure 1:** The behaviour of sign-trackers and goal-trackers differed during the autoshaping sessions. (**A, C**) Number of magazine entries (‘nosepokes’) during lever presentation for each autoshaping session, for rats in Experiment 1 (**A**) and Experiment 2 (**C**). Goal-trackers showed greater numbers of magazine entries than sign-trackers (indicated by the blue asterisks), and sign-trackers made more magazine entries that untrained controls (denoted by the line and $$ symbols). (**B, D**) Number of lever approaches made by rats in Experiment 1 (**B**) and Experiment 2 (**D**). Main effect of Group. * *p* < .05, $$ *p* < .01. Values represent the mean ±/-SEM. Group sizes for A and B: controls, n = 10; goal-trackers, n = 15; sign-trackers, n = 11. Group sizes for C and D: controls, n = 10, goal-trackers, n = 17; sign-trackers, n = 6.
